# Pentraxin-2 is Associated with Renal Fibrosis in Patients Undergoing Renal Biopsy

**DOI:** 10.6061/clinics/2020/e1809

**Published:** 2020-10-21

**Authors:** Taner Basturk, David Ojalvo, Emrah Erkan Mazi, Nuri Baris Hasbal, Ayse Aysim Ozagari, Elbis Ahbap, Tamer Sakaci, Yener Koc, Mustafa Sevinc, Abdulkadir Unsal

**Affiliations:** I University of Health Sciences, Sisli Hamidiye Etfal Teaching and Research Hospital, Istanbul, Turkey; II Vardcentralen Wisby Soder, General Medicine, Gotland, Sweden.; III Sisli Hamidiye Etfal Teaching and Research Hospital, Department of Nephrology, Istanbul, Turkey; IV Hakkari State Hospital, Department of Nephrology, Hakkari, Turkey; V Sisli Hamidiye Etfal Teaching and Research Hospital, Department of Pathology, Istanbul, Turkey; VI Cumhuriyet University, Faculty of Medicine, Department of Internal Medicine, Division of Nephrology, Sivas, Turkey

**Keywords:** Pentraxin, Renal Fibrosis, Chronic Kidney Disease

## Abstract

**OBJECTIVES:**

Progressive renal disease is characterized by histological changes in the kidney and fibrosis is a common outcome. Renal biopsy is the only diagnostic tool to evaluate these histopathological changes. Pentraxin-2 (PTX-2) is an anti-inflammatory constitutive plasma protein associated with the innate immune system. Recently, as a biomarker, the circulating level of PTX-2 is shown to be decreased in chronic fibrotic diseases. In this study, we aimed to investigate the relationship between renal fibrosis severity and serum PTX-2 levels in patients undergoing renal biopsy.

**METHODS:**

This cross-sectional study included 45 patients and 16 healthy individuals (HIs). The severity of renal fibrosis was evaluated according to the Banff and Sethi scoring systems by the same pathologist. PTX-2 was measured by an enzyme-linked immunosorbent assay and compared with the demographical, clinical, biochemical, and histopathological data of the patients and HIs.

**RESULTS:**

PTX-2 levels were lower in the biopsy group than in the HI group (*p*=0.12). Patients with moderate renal fibrosis had significantly lower serum PTX-2 levels than those in patients with minimal and mild fibrosis (*p*=0.017 and *p*=0.010, respectively). PTX-2 concentrations were correlated with serum albumin (r=0.30, *p*=0.016), and were negatively correlated with serum creatinine levels (rho=-0.42, *p*=0.01) and body mass index (r=-0.32, *p*=0.011).

**CONCLUSIONS:**

The results indicated that PTX-2 levels are significantly lower in patients with renal fibrosis than HIs, and declining further in patients with severe fibrosis.

## INTRODUCTION

Chronic kidney disease (CKD) has been a growing health-care problem with increasing prevalence. More than 10% of the population in developed countries is considered to have CKD, with a variety of etiologies such as hypertension, diabetes, glomerulonephritis, and so on (1). The renal parenchyma loses its function because of the immoderate deposition of extracellular matrix components, resulting in renal fibrosis. This kind of fibrosis can affect all histological components of the kidney; it is specifically termed as arteriosclerosis/perivascular fibrosis in the vasculature, interstitial fibrosis in the tubulointerstitium, and glomerulosclerosis in the glomeruli. The degree of tubulointerstitial fibrosis and tubular atrophy in histopathology is well correlated with renal outcomes (2).

Pentraxin-2 (PTX-2), also named as serum amyloid P component, is a constitutive, anti-inflammatory plasma protein associated with the innate immune system. Recently, the circulating level of this protein was shown to be decreased in chronic fibrotic diseases (3). PTX-2 has a unique binding profile, suggesting that it may accumulate at injury sites and helps removing damaged tissue in a non-phlogistic manner. In some fibrotic diseases such as nonalcoholic steatohepatitis, end-stage renal disease, and idiopathic pulmonary fibrosis, continuous generation and utilization of PTX-2 may result in decreasing serum PTX-2 levels (4-6). In this study, we aimed to determine the relationship between PTX-2 levels and the presence or severity of renal fibrosis in patients undergoing percutaneous renal biopsy.

## MATERIAL AND METHODS

### The study group

This cross-sectional study was performed at the Sisli Hamidiye Etfal Teaching and Research Hospital between April 2016 and April 2017 in Istanbul, Turkey. The study included 45 patients who were older than 18 years and had an indication for renal biopsy (Biopsy Group; BG) along with 16 healthy individuals (HIs; for serum parameters only). Informed consent was obtained from all subjects and the study protocol was approved by the Hospital’s Ethical Committee.

The indications assessed by a nephrologist for renal biopsy included acute kidney injury, non-nephrotic proteinuria, proteinuria with coexisting hematuria, isolated hematuria, nephrotic syndrome, and progressive CKD (eGFR≤60 mg/min per 1.73 m^2^). Proteinuria at more than 3.5 g/day and serum albumin below 3.0 g/L were defined as nephrotic syndrome. Non-nephrotic proteinuria was defined as proteinuria lower than 3.5 g/day or proteinuria with concomitant hematuria. Biopsy indications for patients with type 2 diabetes including unexplained rapid worsening of renal function, microscopic hematuria, and proteinuria without diabetic retinopathy. Patients who were clinically suspected for non-diabetic renal disease were selected, examined, and a biopsy was performed if a patient fulfilled the above-mentioned criteria.

Patients who had a small hyperechoic kidney (<9 cm), solitary kidney, multiple and bilateral cysts or renal tumors, anatomical kidney anomalies, active renal, perirenal, or biopsy site infection, malignancy, severe resistant hypertension, or uncontrollable bleeding disorder were excluded. Medications altering the coagulation cascade were withheld until an appropriate time before the procedure. Patients in the biopsy group were evaluated using ultrasonography by an experienced radiologist for kidney biopsy eligibility. The biopsies were performed by an experienced nephrologist or interventional radiologist. After the biopsy, all patients were kept under observation in the hospital for a day in case of complications.

### Laboratory tests

On the day of the kidney biopsy, fasting serum samples for biochemical evaluation were obtained at 08:00 a.m. in all cases. Values of laboratory parameters including complete blood cell counts and serum levels of creatinine, albumin, C-reactive protein (CRP), C3, and C4 were measured using standard enzymatic procedures. Proteinuria was quantified based on 24-h urinary protein excretion measurements. The Chronic Kidney Disease Epidemiology Collaboration (CKD-EPI) equation was used to determine the estimated glomerular filtration rate (eGFR). The severity of kidney failure was estimated according to the Kidney Disease Improving Global Outcomes (KDIGO) guidelines. The body mass index (BMI) was defined as the weight in kilograms/(height in meter)^2^. Proteinuria was excluded in the control group by examining a random morning urine sample.

Blood samples for PTX-2 quantification were obtained on the same day when the patients underwent percutaneous renal biopsy. The sera were kept at -80°C until use. The levels of PTX-2 were determined by enzyme-linked immunosorbent assay (ELISA) (Invitrogen, Grand Island, New York, USA) as per the manufacturer's recommendations.

### Pathological evaluation

The biopsy specimens of the patients were examined by a single experienced pathologist using light and immunofluorescence microscopy. Routine staining analyses, including hematoxylin and eosin, periodic Schiff-methenamine, periodic acid-Schiff reagent, and Masson trichrome staining, were performed. Congo red and methyl violet staining analyses were also performed for samples from some patients. Immunofluorescence staining was performed for IgA, IgG, IgM, C3, C4, C1q, and kappa/lambda light chains.

Interstitial fibrosis and tubular atrophy were scored according to the Banff 97 criteria, with scores from 0 to 3 based on the percentage of cortical parenchyma involved (7). A total renal chronicity score, recently proposed by Sethi et al., was also calculated. Arteriosclerosis/arteriolosclerosis (CV), global and segmental glomerulosclerosis (GS), interstitial fibrosis (IF), and tubular atrophy (TA) were evaluated. The CV was scored from 0 to 1, GS, from 0 to 3, IF, from 0 to 3, and TA, from 0 to 3. The total renal chronicity score was obtained by summing all the scores to stage the overall severity of the chronic lesions as minimal (0-1 total score), mild (2-4 total score), moderate (5-7 total score), and severe (≥8 total scores) (2).

### Statistical analysis

SPSS 21.0 for Windows was used for statistical analysis. Descriptive statistics were presented as number and percentage for categorical changes: as average, standard deviation, minimum, maximum, and median for numerical variations. When the numerical variations maintained the normal distribution conditions, the two independent groups were compared using the Student’s t-test, and when the numerical variations did not maintain the normal distribution conditions, three or more groups were compared using the Kruskal-Wallis test. The study group analysis was performed with Mann Whitey U test and interpreted with Bonferroni correction. The ratios in the groups were compared using Chi-Square analysis. As the relations between numerical variations did not meet the parametrical test condition, Spearman Correlation analysis was used. *p*<0.05 was considered statistically significant.

## RESULTS

This study included 45 patients in the Biopsy Group (BG) and 16 HIs. Their demographic, clinical, and laboratory data are shown in Table 1. The BG had older age, higher BMI, and higher diastolic pressure (Table 1). As expected, lower eGFR and serum albumin levels, and higher serum urea, serum creatinine, and serum C-reactive protein levels were detected in the BG group than in the HIs group. The serum PTX-2 levels were significantly lower in the BG group (*p*=0.012).

The stage of kidney failure was determined for each subject. The numbers of patients with stages 1-5 of kidney failure were: 9 (20%), 16 (35.6%), 8 (17.8%), 6 (13.3%), and 6 (13.3%), respectively. There was no correlation between the stage of kidney failure and PTX-2 level. The pathological diagnosis of the patients was as follows: focal segmental glomerulosclerosis (FSGS) (n=18), IgA nephropathy (n=9), membranous glomerulonephritis (MGN) (n=6), amyloidosis (n=3), tubulointerstitial nephritis (n=3), lupus nephritis (n=2), myeloma cast nephropathy (n=1), cholesterol crystal embolism (n=1), membranoproliferative glomerulonephritis (n=1), and diabetic glomerulosclerosis (n=1).

**Figure 1:**
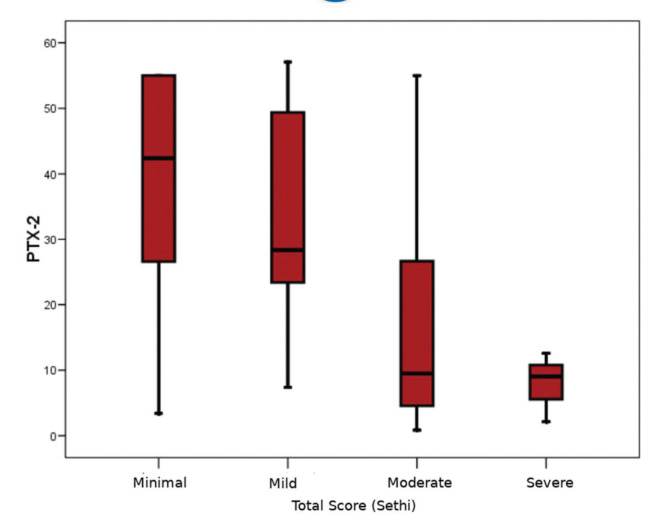
Decrease in serum PTX-2 levels relative to the severity of renal fibrosis.

The pathological evaluation of biopsy specimens is summarized in Table 2. The severity of fibrosis was reported as minimal (n=14; 31.1%), mild (n=17; 37.8%), moderate (n=11; 24.4%), and severe (n=3; 6.7%). The mean serum PTX-2 levels of the patients with minimal, mild, moderate, and severe renal fibrosis were 38.0±18.1 ng/mL, 32.8±17.1 ng/mL, 16.3±16.6 ng/mL, and 7.9±5.3 ng/mL, respectively. In the subgroup analysis, patients with moderate renal fibrosis had significantly lower serum PTX-2 levels than those with minimal and mild fibrosis (*p*=0.017 and *p*=0.010, respectively) (Figure 1). There was no difference between the PTX-2 levels in patients with minimal and mild fibrosis. As the number of patients with severe fibrosis (n=3) was very low, subgroup analysis could not be performed. Univariate correlations between the PTX-2 levels and pathological findings are shown in Table 3. Although there were small numbers of patients with each pathological diagnosis, no significant differences were found between the median PTX-2 levels and each diagnosis type. Based on the Spearman's rank correlations (Rho), PTX-2 concentrations were correlated with serum albumin levels (r=0.30, *p*=0.016), and negatively with serum creatinine (rho=-0.42, *p*=0.01) and BMI (r=-0.32, *p*=0.011) (Table 3).

## DISCUSSION

Pentraxins such as pentraxin-2 (known as serum amyloid P, SAP) regulate various features of the innate immune system. PTX-2 increases the migration of immune-regulatory macrophages, suppresses the differentiation of monocyte-derived fibroblast-like cells, and inhibits neutrophil adhesion to extracellular matrix proteins (3).

A prognostic marker for CKD has been explored for years. A large number of serum and urinary molecules such as NGAL, KIM-1, MCP-1, MMP-9, interleukin-18, clusterin, MMP-9, TIMP-1, Procollagen I alpha 1, FGF-23, suPAR, as well as PTX-2 are under investigation to predict CKD progression (8,9). All these biomarkers have been studied for predicting CKD progression in cohorts of patients with different stages of chronic kidney disease and with various etiologies (proteinuric and non-proteinuric glomerulonephritis, diabetic, hypertensive, and polycystic kidney diseases). However, the available data are insufficient to draw final conclusions. Thus, further studies with larger cohorts and longer follow-up periods are required.

Accumulation of PTX-2 at the site of fibrosis has been shown in animal models (10). PTX-2 suppresses the differentiation of monocytes into fibrotic and inflammatory macrophages and fibrocytes, while supporting their differentiation into regulatory macrophages (11). Therefore, epithelial healing and decomposition of scarring and inflammation are promoted. At fibrotic sites, continuous production and consumption of PTX-2 may result in reduced levels of the protein in fibrotic diseases, as seen in idiopathic end-stage renal disease, pulmonary fibrosis, and nonalcoholic steatohepatitis (5,12,13).

Castaão et al. performed a study about the anti-fibrotic effects of PTX-2 and the potential mechanisms underlying its action. Human SAP (hSAP) was found to be a natural inhibitor of fibrosis during kidney injury via the downregulation of fibrotic collagen gene transcription. On the contrary, fibrocytes were infrequently seen in injured kidneys following treatment with hSAP, proving that fibrocytes do not play a major role in fibrosis. A correlation between loss of kidney function and lower hSAP concentrations has also been reported in mice (5).

Another study focused on recombinant PTX-2 treatment in mice deficient in the kidney microvascular basement membrane protein collagen type IV (α)3 (*Col4a3^-1^* ^-^mice) (6). This protein plays a key role in human Alport Syndrome (14). The mutant mice that received intraperitoneal recombinant human PTX-2 (rhPTX-2) injections for 9 weeks showed an improvement in their lifespan by >20%. rhPTX-2-treated mice showed reduced glomerulosclerosis, preserved podocyte numbers in glomeruli, approximately 40% less tubule injury, and attenuated interstitial fibrosis. Nakagawa et al. also reported decreased numbers of macrophages in the diseased kidneys, enhancement of IL-10 production, and downregulation of secreted proteins associated with fibrosis following rhPTX-2 administration. The transcriptional regulator c-Jun and its activator protein-1 binding partners were identified in this study as the main target for rhPTX-2 function (6).

All the subjects enrolled in our study were indicated for renal biopsy, and fibrosis was found ranging from the minimal to severe grade in all the specimens. Thus, in agreement with animal studies, PTX-2 levels were lower in patients with renal fibrosis than in HIs. We also found significantly lower PTX-2 levels in patients with moderate renal fibrosis than in those with minimal and mild fibrosis. As the number of patients with severe renal fibrosis was small, we could not perform a subgroup analysis; however, it is noteworthy that this group had the lowest PTX-2 level.

We also found that the level of PTX-2 was negatively correlated with the BMI. Although the sample size was small, there were no associations between BMI and the pathological diagnosis of patients. However, it is expected that patients with FSGS may have higher BMI. Obesity-associated FSGS is a well-known phenomenon, and is also called obesity-related glomerulopathy. The pathological components of this disease include glomerulomegaly, FSGS (particularly the perihilar variant), reduced glomerular and podocyte density, and diabetoid changes (15). Isolated proteinuria characterized as stable or slowly progressive is the cornerstone clinical manifestation of this disease. Further, there is a correlation between proteinuria and the number of glomeruli showing FSGS. Therefore, higher BMI-induced glomerulomegaly, podocyte injury, FSGS, and fibrosis may explain the negative association between the PTX-2 levels and BMI.

Based on the potential role of PTX-2 in wound healing and fibrosis, an increasing number of studies are testing PTX-2 as a therapeutic agent. For instance, Blink et al. performed a study of 21 patients with idiopathic pulmonary fibrosis (IPF) and did not report any benefit of PTX-2 (PRM-151) administration (16). Raghu et al. designed a similar multicenter study with 117 patients and reported a slower decline in lung function over 28 weeks, as infusions of human PTX-2 were administered to patients with IPF (12). We believe that PTX-2, as a potential treatment strategy for patients with renal fibrosis, will be the focus of human trials in the near future.

This study has a few limitations; it was a single center study with a small cohort size and a limited number of unmatched healthy controls. As the number of patients with severe renal fibrosis was small, we could not perform a subgroup analysis. Further, because there were only a few patients for each pathological diagnosis, we could not evaluate the difference between the serum PTX-2 levels in various specific diseases.

In conclusion, we report the first study in human subjects investigating the level of PTX-2 in patients who underwent renal biopsy. This study demonstrated that PTX-2 levels are lower in patients with renal fibrosis than HIs and declining further in patients with more severe fibrosis. Therefore, PTX-2 may be a biomarker of fibrosis, and could be used as a prognostic indicator to guide treatment. However, extensive additional studies are required to firmly establish the relationship between serum PTX-2 levels and renal fibrosis.

## CONFLICTS OF INTEREST

The article was presented as an oral presentation at the 36^th^ National Congress of Nephrology, Antalya, Turkey on 19^th^ October 2019.

## AUTHOR CONTRIBUTIONS

Basturk T and Ahbap E contributed in research idea and study design. Ojalvo D, Mazi EE, Ozagari AA and Hasbal NB contributed with data acquisition, analysis and interpretation. Ojalvo D and Ahbap E were responsible for statistical analysis. Basturk T, Ahbap E, Sakaci T, Koc Y, Sevinc M, Unsal A contributed to supervision or mentorship.

## Figures and Tables

**Table 1:** Demographic, clinical, and laboratory data of the Study Group (unless otherwise mentioned, all variables are mean (SD): mean±standard deviation).

	Biopsy Group	HIs Group	*p*
Number (n)	45	16	NA
Age, median (min-max)	45.6 (18-79)	29.5 (18-63)	<0.001
Sex, female (%)	33	68.8	<0.001
BMI, kg/m^2^	25.4±4.7	21±2.1	<0.001
SBP, mmHg, me	127.8±16.2	119.3±8.7	0.131
DBP, mmHg	78.2±7.7	65.6±5.4	<0.001
Proteinuria, g/day	3.42±3.7	NA	NA
eGFR, mL/min/m^2^	60.7±35.4	118.18±12.1	<0.001
Serum Urea, mg/dL	58.7±41.5	23.4±5.4	<0.001
Serum Creatinine, mg/dL	1.99±1.73	0.70±0.13	<0.001
Serum Albumin, g/dL	3.63±0.74	4.68±0.38	<0.001
WBC, /mm^3^	8574.0±3193.6	6890.6±2207.4	<0.001
CRP, mg/L	9.6±17.6	2.5±1.3	<0.001
PTX-2, ng/mL	28.7±19.2	43.2±13.6	0.012

Abbreviations: BMI, body mass index; SBP, systolic blood pressure; DBP, diastolic blood pressure; eGFR, estimated glomerular filtration rate; WBC, white blood cells; CRP, C-reactive protein; PTX-2, pentraxin-2; NA, not applicable.

**Table 2:** Pathological evaluation of the biopsy specimens according to Banff and Sethi scoring.

Scoring System	Quantitation	n	%
IF (Banff)	0	17	37.8
	1	18	40.0
	2	8	17.8
	3	2	4.4
TA (Banff)	0	7	15.6
	1	28	62.2
	2	8	17.8
	3	2	4.4
GS (Sethi)	0	14	31.1
	1	11	24.4
	2	17	37.8
	3	3	6.7
IF (Sethi)	0	18	40.0
	1	17	37.8
	2	8	17.8
	3	2	4.4
TA (Sethi)	0	20	44.4
	1	15	33.3
	2	8	17.8
	3	2	4.4
CV (Sethi)	0	28	62.2
	1	17	37.8

Abbreviations: GS, Global and segmental glomerulosclerosis; TA, tubular atrophy; IF, interstitial fibrosis; CV, arteriosclerosis/arteriolosclerosis.

**Table 3:** Univariate correlations between PTX-2 levels and clinical characteristics, pathological findings, and investigated biomarkers.

	PTX-2	
	*R*	*p*
Age	-0.130	0.319
BMI	-0.325	0.011
SBP	-0.065	0.632
DBP	-0.276	0.038
eGFR	0.231	0.127
Proteinuria	-0.247	0.102
Stage of kidney failure	-0.259	0.085
Biopsy		
IF (Banff)	-0.352	0.018
TA (Banff)	-0.350	0.018
GS (Sethi)	-0.398	0.007
IF (Sethi)	-0.350	0.018
TA (Sethi)	-0.341	0.022
CV (Sethi)	-0.131	0.392
Total Score (Sethi)	-0.399	0.007
Urea	-0.210	0.104
Creatinine	-0.426	0.001
Albumin	0.307	0.016
WBC	-0.077	0.557
CRP	-0.157	0.226

Abbreviations: BMI, body mass index; SBP, systolic blood pressure; DBP, diastolic blood pressure; eGFR, estimated glomerular filtration rate; WBC, white blood cells; CRP, C-reactive protein; PTX-2, pentraxin-2.
